# Transcriptomics integrated with metabolomics reveals the defense response of insect-resistant *Zea mays* infested with *Spodoptera exigua*

**DOI:** 10.1016/j.heliyon.2025.e42565

**Published:** 2025-02-08

**Authors:** Qiulan Luo, Fangmeng Duan, Wenwen Song

**Affiliations:** a School of Life Sciences and Food Engineering, Hanshan Normal University, Chaozhou, 521041, People's Republic of China; b College of Plant Health and Medicine, Qingdao Agricultural University, Qingdao, 266109, People's Republic of China

**Keywords:** Maize, *Spodoptera exigua*, Transcriptomic and metabolic analysis

## Abstract

Maize (*Zea mays*) is one of the most important cereal crops worldwide. Insect control through host plant resistance plays an important part in improving both yield and quality of maize. *Spodoptera exigua* is a common insect pest causing destructive damages to maize. To comprehensively understand molecular mechanism of maize defense against *S. exigua*, integrated transcriptomics and metabolomics analyses were conducted in the insect-resistant maize inbred line CML139 infested with *S. exigua* for 24 h. 9845 differentially expressed genes and 34 significantly changed metabolites were identified in infested leaves. Maize transcriptional response to *S. exigua* infestation involved in genes encoding enzymes in biosynthetic process (ribosome, glycerolipid, glycerophospholipid metabolism), genes in valine, leucine and isoleucine degradation, phenylpropanoid pathway and transcription factors. By metabolism analysis, accumulations of amino acids, organic acids, phenylpropanoids and benzoxazinoids (Bxs) were significantly enhanced, with the exception of salicylic acid (SA) and jasmonic acid (JA). The integrated analysis of transcriptomic and metabolic data demonstrated that both transcripts and metabolites involved in phenylpropanoid and Bxs biosynthesis were differentially modulated in *S. exigua* infested leaves. This study is valuable in understanding the complex mechanism of interaction between plants and insect herbivores and provide a potential strategy to maize pest control.

## 1 Introduction

Plants have evolved a variety of defense mechanisms to protect themselves against herbivores attack [1,2]. Plants respond to damage by herbivory through large-scale changes in gene expression and metabolism [[3], [4], [5], [6], [7], [8], [9]]. Herbivorous insect infestation can alter expression levels of related genes and promote the biosynthesis of numerous metabolites in maize through complex molecular mechanisms at transcriptional and translational levels [10]. With the rapid development of omics technology, transcriptomics and metabolomics provide valuable opportunities to explore the molecular mechanisms of plants infested by herbivorous insects [11,12]. The integration of transcriptomics and metabolomics will identify more applicable resistance genes and demonstrate the correlation between genes and metabolites in plants when subjected to pest infestation [12].

Maize (*Zea mays*) is one of the most important cereal crops worldwide. Both yield and quality of maize are severely threatened by various insect pests. Beet armyworm (*Spodoptera exigua*) is a major pest in agricultural production with a wide host range that can cause serious economic losses mainly through larvae crop leaf consumption [6]. Metabolite profiling revealed that a series of defence-related plant secondary metabolites, including benzoxazinoids (Bxs), chlorogenic acid and maysin, was induced in response to *S. exigua* feeding [13].

Salicylic acid (SA) and jasmonic acid (JA) derivatives are major plant hormones, play important roles in pest defense by modulate toxic metabolites biosynthesis [6,[14], [15], [16], [17]]. JA and its derivatives, methyl jasmonate and jasmonate-isoleucine, have been considered the most effective hormones in pest defense [17]. Also genes sequenced in the B73 maize genome encoding enzymes involved in jasmonate biosynthesis have been well illustrated [10].

Previous researches demonstrated that the contents of Bxs were strongly increased in maize by herbivore attack, including 2,4-dihydroxy-7-methoxy-1,4-benzoxazin-3-one glucoside (DIMBOA-Glc), 2,4-dihydroxy-7,8-dimethoxy-1,4-benzoxazin-3-one glucoside (DIM2BOA-Glc), 2-hydroxy-4,7-dimethoxy-1,4-benzoxazin-3-one glucoside (HDMBOA-Glc), 6-methoxybenzoxazolin-2(3H)-one (MBOA) and 2-hydroxy-4,7,8-trimethoxy-1,4-benzoxazin-3-one glucoside (HDM2BOA-Glc) [13,[18], [19], [20], [21], [22]]. However, changes in gene expressions and metabolite contents of Bxs in insect-resistant maize after *S. exigua* infestation remain unknown. Besides, only partial chemical compounds involved in pest defense of maize leaves have been identified, it is likely that many as yet unknown defensive metabolites need be revealed. Therefore, an exploration of the molecular mechanism underlying maize resistance to *S. exigua* is required to cultivate resistant maize varieties and develop effective pest control measurements.

In previous studies, the multi-omics researches on maize plants were mostly investigated using inbred line B73 because it has an available genome sequence [23]. By integrating transcriptomics and metabolomics analyses, the genetic basis for maize defense against insect herbivory is beginning to be elucidated [10,13,24]. For example, Tzin et al. [13] examined the dynamic responses of maize in response to caterpillar feeding. In B73, there were two major defense related pathways, benzoxazinoid and jasmonic acid biosynthesis, similar results have been obtained in other studies [24,25]. Whereas, B73 which is highly susceptible to insects, was used as plant material in the above researches. B73 is highly susceptible to insects. There has been no lack of studies related with B73 responses to damage caused by herbivory insect. However, the molecular mechanism underling the insect-resistant response of maize remains unknown. Here, this study will enhance the understanding of the molecular mechanism underlying the resistant response of maize against caterpillar feeding.

By integrating transcriptomics and metabolomics analyses, the genetic basis for maize defense against insect herbivory is beginning to be elucidated [10,13,24]. For example, Tzin et al. [13] examined the dynamic responses of maize in response to caterpillar feeding and identified two major defense related pathways, Bxs and JA biosynthesis; similar results have been obtained in other studies [24,25]. The inbred line B73, which is highly susceptible to insects, was used as plant material in the above researches. However, the molecular mechanism underlying *S. exigua* defense in insect-resistant maize remains unexplored.

CML139, as a highly insect-resistant maize inbred line, which is developed from the International Maize and Wheat Improvement Center (CIMMYT) materials for tropical borer resistance [26]. CML139 is a subtropical, yellow-red, semi-flint line with intermediate maturity. Compared to insect-susceptible line Ki3, CML139 decreased leaf damage rating by 28.7 % [26]. In our study, transcriptomics integrated with metabolomics analyse was combined to reveal the delicate response of insect-resistant maize seedlings CML139 exposed to *S. exigua* caterpillar feeding in order to investigate the resistant molecular mechanisms of plant-herbivore interaction. Our results will provide patterns of gene expression and metabolite profile, which will enhance understanding of resistant maize defense mechanisms to *S. exigua* infestation.

## 2 Materials and methods

### 2.1 Plants and growth conditions

*Zea mays* inbred line CML139 was used as plant material. After germination, each seed was sown in 6.7 cm × 7.0 cm (220 cm^3^) plastic pots filled with sterilized vermiculite. Seedlings were placed into growth chambers at 25 °C, 60 % relative humidity, 36 μmol m^−2^·s^−1^ light intensity, with a 16:8 h/light: dark photoperiod and irrigated with Hoagland plant nutrient solution until reaching the three-leaf stage.

### 2.2 Caterpillar bioassay

*S. exigua* larvae were obtained from Hailir Pesticides and Chemicals Group (Qingdao, China). Each maize plant was infested with three third instar caterpillars on the third leaf. These caterpillars were immediately caged in a micro-perforated polypropylene bag to keep them on the third leaf. The second leaf was harvested at 0, 1, 3, 6, 12, 24, and 48 h after infestation of the third leaf. The second leaves of healthy seedlings, which were notinfested, served as the control. All samples were frozen with liquid nitrogen and stored at −80 °C for transcriptomics and metabolomics analyses. Each treatment consisted of six plants, was performed in three biological replicates in which there were three technical replicates.

### 2.3 Transcriptome sequencing and analysis

Total RNA was extracted from each sample using RNeasy Plant Mini Kit (Qiagen, Germany) according to the manufacturer's instructions. RNA quality and quantity were examined using an Agilent 2100 Bioanalyzer (Agilent, CA, USA). A first-strand cDNA was synthesis by a PrimeScript™ RT reagent kit (Takara, Kyoto, Japan). qRT-PCR was carried out in an Agilent StrataGene M3005 (Agilent, Santa Clara, CA, USA) using a SYBR Premix Ex Taq Kit (Takara, Kyoto, Japan). Sampling time of transcriptome sequencing was dependent upon when the relative expression level of the chitinase gene (GenBank accession: MG017379.1) and *MPI* gene (Maize Proteinase Inhibitor, *MPI*, GenBank accession: X78988) in infested maize leaves exhibited the highest transcription level, whose transcript abundances were significantly increased in response to herbivory feeding [27,28]. The transcript level of the above two genes were detected by qRT-PCR. Premix SYBR green qRT-PCR master mix (TaKaRa Corp) and an ABI 7500 (Applied Biosystems) were used for qRT-PCR. The specific primers were designed as chitinase-F: 5′-CCCCGAGTCGCTCTTCAACC-3′ and chitinase-R: 5′-CGGCGTCATCCAGAACCAG-3’; MPI-F: 5′-ACAACCAGCAGTGCAACAAG-3′ and MPI-R: 5′-GAAGATGCGGACACGGTTAG [29]. The glyceraldehyde-3-phosphate dehydrogenase gene (*ZmGAPc*, GenBank: X07156) served as an internal control to determine the relative transcript levels of the chitinase gene and *MPI* gene. The comparative threshold cycles were applied as follows: 98 °C for 5 min, 35 cycles of 98 °C for 10 s, 62 °C for 10 s, and 72 °C for 25 s. At the end of the PCR cycles, the products were subjected to melt curve analysis to verify PCR amplification specificity. Each gene expressed in control plants at 0 h was normalized to 1 and treatment expression was calculated relative to this level. The relative expression level was measured via the ^ΔΔ^Ct method. qRT-PCR was performed in three biological replicates and each reaction was processed three times.

A total of 2 μg RNA was used for library construction. The cDNA library was separately constructed for subsequent sequencing (RNA-seq) on an Illumina Hiseq 4000 platform (Illumina, San Diego, CA, USA). Paired-end raw reads were then processed by removal of adapters and low-quality sequences using SOAPnuke software [30]. RNAseq analysis was conducted using the maize genome version B73 AGP v3.22 as a reference. Reads were mapped with TopHat2 (Kim et al., 2013) followed by expression analysis using the Cuffdiff package (Trapnell et al., 2012) version 2.2.1. Transcripts exhibiting at least one fragment per kilobase of exon per million fragments (FPKM) of transcript in three replicates were kept for differentially expressed gene detection. Genes whose expression differed by fold change >2 or <0.5 and *P*-value <0.05 were identified as differentially expressed genes (DEGs). GO analysis was carried out with the GSEABase (gene set enrichment analysis base) package from BioConductor. Pathway analysis was performed to identify significant pathways of DEGs according to the Kyoto Encyclopedia of Gene and Genomes (KEGG).

### 2.4 Metabolite extraction

Six replicates, each consisting of six pooled plants obtained from two independent groups (treatment and control) were subjected to metabolomics analysis. Metabolites were extracted from 100 mg of leaves according to a previously described protocol [31]. Briefly, leaves were chilled with liquid nitrogen and ground to powder by a high flux organization grinding apparatus (70 Hz for 1 min), then extracted with 1000 μL methanol (pre-cooled at −20 °C) for 30 s. Samples were placed into a sonicator. The ultrasonic extraction conditions were as following: ultrasonic temperature 25 °C, ultrasonic time 30 min and ultrasonic power 100 kHz. Then, 750 μL chloroform (pre-cooled at −20 °C) and 800 μL deionized water (pre-cooled at 4 °C) were added and vortexed for 60 s. After centrifugation for 10 min at 10 000×*g*, the supernatant was transferred into a new centrifuge tube, then air-dried by vacuum concentration. The dried residue was re-dissolved in 250 μL 50 % methanol aqueous solution (pre-cooled at 4 °C) and filtered by a 0.22 μm membrane for LC-MS analysis.

### 2.5 LC-MS analysis

LC-MS analysis was performed using an Acquity UPLC system (Waters Corp., Watertown, MA, USA) equipped with an HSS T3 column (150 × 2.1 mm, 1.8 μm). The gradient elution of analytes was carried out with 0.1 % formic acid in water (A) and 0.1 % formic acid in acetonitrile (B) at a flow rate of 0.25 mL/min 6 μL injection of each sample was collected after equilibration. An increasing linear gradient of solvent B (v/v) was used as follows: 0–1 min, 2 % B; 1–11 min, 2%–50 % B; 11–17 min, 50%–98 % B; 17–18 min, 98 % B; 18–18.5 min, 98%–2% B; 18.5–21 min, 2 % B.

The LC-MS experiments were executed on a Thermo LTQ-Orbitrap XL mass spectrometer with a capillary voltage and spray shield of 4.5 kV. Sheath gas and auxiliary gas were set at 40 and 15 arbitrary units, respectively. Spectra were acquired over the *m*/*z* 50–1200 range. The normalized collision energy was 30 eV.

### 2.6 Integrated analysis of transcriptomics and metabolomics data

The exons, introns, and intergenic spacers were identified using HISAT software through mapping to *Z. mays* genome data, version B73 RefGen v2. MBROLE 2.0 software was utilized to build networks of genes and metabolites from transcriptome and metabolomic data. The metabolites were mapped to KEGG pathways (KEGG compound IDs). Only metabolites with q-values less than 0.05 and genes in significantly enriched pathways (*P* < 0.05) were selected for further correlation analysis through correlation coefficients. Metabolites and genes were integrated in the same KEGG pathway.

### 2.7 Extraction and quantiﬁcation of endogenous JA and SA

Extraction and quantiﬁcation of JA and SA were performed as described previously [32]. In brief, JA and SA were isolated by extraction reagents, after dried and dissolved in 0.5 mL methanol, filtered and used for HPLC analysis.

### 2.8 Quantitative real-time PCR analysis

To verify gene expression during pest defense, a total of ten DEGs were randomly selected and their transcriptional profiles were examined by qRT-PCR. Genes and primer pairs were shown in Supplementary Table S1. The expression patterns of the ten selected genes relative to a housekeeping gene (*ZmGAPc*) were confirmed by qRT-PCR using an independently generated cDNA library. The RNA extraction and qRT-PCR were performed as mentioned above.

### 2.9 Statistical analysis

Data including gene expression levels, endogenous JA and SA content were conducted on statistical analysis using the SPSS statistical software (ver.16.0, SPSS Inc., Chicago, IL, United States) by one-way analysis of variance followed by Tukey's test. Error bars represent standard deviations (SD) with diﬀerent letters at *P* < 0.05 signiﬁcant level.

## 3 Results

### 3.1 Transcriptomic analysis of maize leaves after *S. exigua* caterpillar feeding

The transcriptional expression levels of both genes related to insect resistance, chitinase and *MPI* expression exhibited the similar trend; lower abundance at the first four time points followed by a significant increased at 12 h. At 24 h after *S. exigua* caterpillar feeding, both of them reached the highest peak; while there was a dramatic reduction of transcriptional abundance of these two genes in the 48 h-infested leaves (Fig. S1). Therefore, we focused our transcriptomic and metabolic assays on the leaves infested for 24 h.

### 3.2 Quality assessment of transcriptomic sequencing

Transcriptomic sequencing of maize leaves with *S. exigua* caterpillar feeding and controls generated 163 679 354 and 141 947 772 raw reads, respectively. All libraries containing more than 96.38 % of the sequence had a quality score above Q20, and the GC percentages were above 57.27 % (Table S2), indicating that all libraries were high quality. A total of 158 160 932 and 136 785 892 clean reads were filtered from the treatment and control groups, respectively. Clean reads had mapping rates above 87.18 %. The transcriptomics data are available from the NCBI database of the Sequence Read Archive project under the accession number SRP160910.

### 3.3 Screening of differentially expressed genes (DEGs)

A total of 21772 unigenes were detected in leaves infested by *S. exigua* (Table S3). Additionally, 9845 DEGs were identified in maize leaves upon insect infestation, including 4641 up-regulated genes and 5204 down-regulated genes (Fig. S2).

### 3.4 Gene ontology (GO) of DEGs

To explore the transcriptional regulatory mechanism of maize response to insects, all DEGs were catalogued according to GO. There were 20 notably enriched GO terms of upregulated DEGs exhibited in Fig. S3A. The GO term representing phospholipid metabolic process (GO: 0006644) was the most significant enrichment, implying that this process played an important role in maize against *S. exigua* attack in order to produce important precursors for biosynthesis of insect-resistant compounds. An enhanced number of DEGs functioning as lipid metabolic process was assembled under the GO term (GO: 0006629), which included 179 genes.

Similarly, biological process still dominated in the top 30 GO terms of downregulated DEGs. Besides, there was distinct difference between the enrichment results of upregulated and downregulated DEGs. In addition to biological process and molecular function, cellular component was also detected to be enriched (Fig. S3B), in which, a large number of DEGs were classified as nitrogen compound metabolic process (GO: 0006807).

### 3.5 KEGG pathway analysis of DEGs

To identify which biochemical pathways were regulated by insect feeding, all DEGs were mapped to the KEGG database. A total of 4668 DEGs were assigned to 126 KEGG pathways. The significantly abundantly represented pathways of upregulated genes were exhibited in Table 1, among which, the most significant enrichment was valine, leucine and isoleucine degradation, followed by glycerolipid and glycerophospholipid metabolism.

**Table 1:** Summary of significantly enriched (*P* < 0.05) pathways associated with differentially expressed genes (DEGs).

Class	Pathway ID	Pathway term	Number of DEGs	*P* Value
Pathways associated with up regulated DEGs	zma00280	Valine, leucine and isoleucine degradation	26	2.55E-05
	zma00561	Glycerolipid metabolism	30	2.76E-05
	zma00564	Glycerophospholipid metabolism	35	5.54E-04
	zma03040	Spliceosome	57	2.33E-03
	zma00072	Synthesis and degradation of ketone bodies	6	8.28E-03
	zma00790	Folate biosynthesis	9	8.87E-03
	zma00760	Nicotinate and nicotinamide metabolism	9	1.36E-02
	zma00071	Fatty acid degradation	16	1.55E-02
	zma04146	Peroxisome	27	1.92E-02
	zma04144	Endocytosis	51	2.30E-02
	zma00860	Porphyrin and chlorophyll metabolism	16	2.75E-02
	zma00600	Sphingolipid metabolism	13	3.94E-02
	zma00310	Lysine degradation	11	4.27E-02
	zma04140	Regulation of autophagy	13	4.90E-02
Pathways associated with down regulated DEGs	zma03010	Ribosome	200	5.22E-20
	zma03008	Ribosome biogenesis in eukaryotes	40	2.20E-03
	zma00670	One carbon pool by folate	11	5.80E-03
	zma01230	Biosynthesis of amino acids	87	1.53E-02
	zma01200	Carbon metabolism	86	2.08E-02
	zma00460	Cyanoamino acid metabolism	18	3.03E-02
	zma03410	Base excision repair	16	3.63E-02
	zma03013	RNA transport	58	4.51E-02
	zma00030	Pentose phosphate pathway	23	4.69E-02

Interestingly, there were two common pathways between upregulated genes and downregulated ones, which were involved in porphyrin and chlorophyll metabolism and folate biosynthesis (One carbon pool by folate in Table 1), implying different numbers in the same family involved in the same pathway might play different roles in resistance to insect infestation.

### 3.6 Transcription factor families in DEGs

The transcriptional abundances of 741 TFs were modulated by insect feeding in maize leaves, which could be classified into 71 TF families (Fig. S4 and Table S4). These TFs mainly include the following families: AP2-EREBP (apetala2ethylene-responsive element binding proteins) (37 genes), bHLH (basic helix-loop-helix) (36 genes), bZIP (basic region/leucine zipper motif) (44 genes), HB (hunchback) (34 genes), MYB (myeloblastosis) (56 genes), NAC (NAM, ATAF1-2, and CUC2) (27 genes), Orphans (34 genes) and WRKY (27 genes). Of these, MYB and bZIP were the two most abundant families, containing 56 and 44 members, respectively.

### 3.7 Alteration in the metabolite signature of maize leaves induced by *S. exigua* infestation

LC-MS metabolic profiling of samples taken from leaves infested for 24 h identified 34 known metabolites from 1913 peaks in the chromatograms (Table S5). Contents of 27 metabolites were notably enhanced and those of the rest were significantly decreased in infested leaves compared to the controls. Among metabolites, amino acids and the secondary metabolites were the most prevalent (35.29 % of the metabolites), followed by organic acids accounting for 17.65 %.

An enrichment analysis of the pathways mediated by these metabolites was carried out, and the significantly enriched pathways were selected for further analysis. The result showed that 27 metabolites with significantly increased contents involved in 31 pathways, among which, 12 pathways (*P*-value <0.05) were remarkably enriched. Besides, biosynthesis and metabolism of amino acids and phenylalanine metabolism were the most enrichment. While, there were only 6 pathways of significant enrichment according to the KEGG analysis for 7 metabolites with significantly decreased contents, such as citrate cycle, C5-Branched dibasic acid metabolism, glyoxylate and dicarboxylate metabolism, metabolic pathways, taurine and hypotaurine metabolism and alanine, aspartate and glutamate metabolism (Table S6).

### 3.8 Integrated analysis of transcriptomics and metabolomics

To reveal the relationship between gene and metabolite alterations during insect infestation, MBROLE2.0 was utilized to visualize and clarify the transcriptomics and metabolomics data. Metabolites and genes were considered integrated when up- or down-expressed metabolites and genes were mapped to the same KEGG pathway. The results comprised 12 up- and 6 down-expressed pathways, including 2911 up-regulated and 1595 down-regulated expressed genes, respectively (Table 2, Table 3). The result demonstrated that both up- and down-expressed pathways shared the common pathways, which included alanine, aspartate and glutamate metabolism and metabolic pathways.

**Table 2:** Summary of significantly enriched (*P* < 0.05) pathway terms associated with up-regulated differentially expressed genes (DEGs).

Pathway ID	Pathway term	Number of DEGs	*P* value
zma00970	Aminoacyl-tRNA biosynthesis	75	6.95E-13
zma00260	Glycine, serine and threonine metabolism	49	1.09E-04
zma00290	Valine, leucine and isoleucine biosynthesis	28	4.01E-04
zma00460	Cyanoamino acid metabolism	41	1.25E-03
zma00360	Phenylalanine metabolism	46	1.75E-03
zma00250	Alanine, aspartate and glutamate metabolism	24	7.11E-03
zma01110	Biosynthesis of secondary metabolites	1038	8.26E-03
zma00400	Phenylalanine, tyrosine and tryptophan biosynthesis	27	8.96E-03
zma00410	beta-Alanine metabolism	31	1.17E-02
zma00280	Valine, leucine and isoleucine degradation	41	2.00E-02
zma00270	Cysteine and methionine metabolism	56	3.58E-02
zma01100	Metabolic pathways	1455	4.23E-02

**Table 3:** Summary of significantly enriched (*P* < 0.05) pathway terms associated with down-regulated differentially expressed genes (DEGs).

Pathway ID	Pathway term	Number of DEGs	*P* value
zma00020	Citrate cycle (TCA cycle)	20	6.41E-06
zma00660	C5-Branched dibasic acid metabolism	32	2.76E-05
zma00630	Glyoxylate and dicarboxylate metabolism	44	7.28E-05
zma01100	Metabolic pathways	1455	3.05E-02
zma00430	Taurine and hypotaurine metabolism	20	4.14E-02
zma00250	Alanine, aspartate and glutamate metabolism	24	4.95E-02

**Fig. 1:**
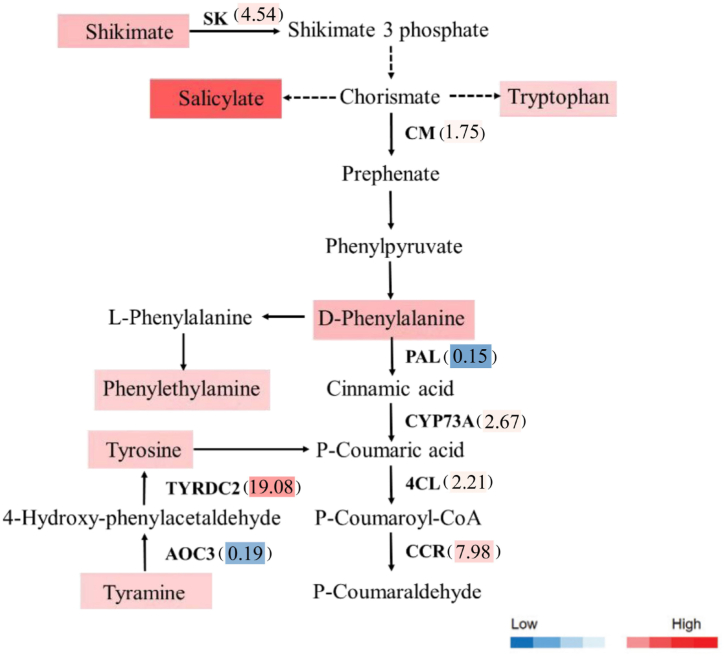
Expression patterns of caterpillar feeding induced genes and metabolites involved in phenylpropanoid biosynthesis. Values in brackets are presented genes expression fold change relative to untreated control. Different colors represent gene expression levels.

To investigate the role of the phenylalanine pathway in the biosynthesis of active compounds in leaves infected by *S. exigua*, an integrated analysis was conducted on the transcript and metabolic levels involving in phenylpropanoid biosynthesis (Fig. 1). Seven metabolites, including shikimate, salicylate, tryptophan, phenylalanine, phenylethylamine, tyramine and tyrosine were induced remarkably in the phenylalanine metabolic pathway. At the same time, 8 genes encoding enzymes involved in phenylalanine metabolism were significantly regulated, which encode shikimate kinase (SK), chorismate mutase (CM), phenylalanine ammonia-lyase (PAL), CYP73A, 4-coumarate-CoA ligase (4CL) and cinnamoyl-CoA reductase (CCR), allene oxide cyclase 3 (AOC3), tyrosine/DOPA decarboxylase 2 (TYRDC2) respectively. It was interesting that when subjected to the insect feeding, the transcriptional abundances of the genes encoding the above enzymes were induced notably, with exception of *PAL* and *AOC3*, whose transcript exhibited a distinct fold reduction (6.73 and 5.31 folds).

**Fig. 2:**
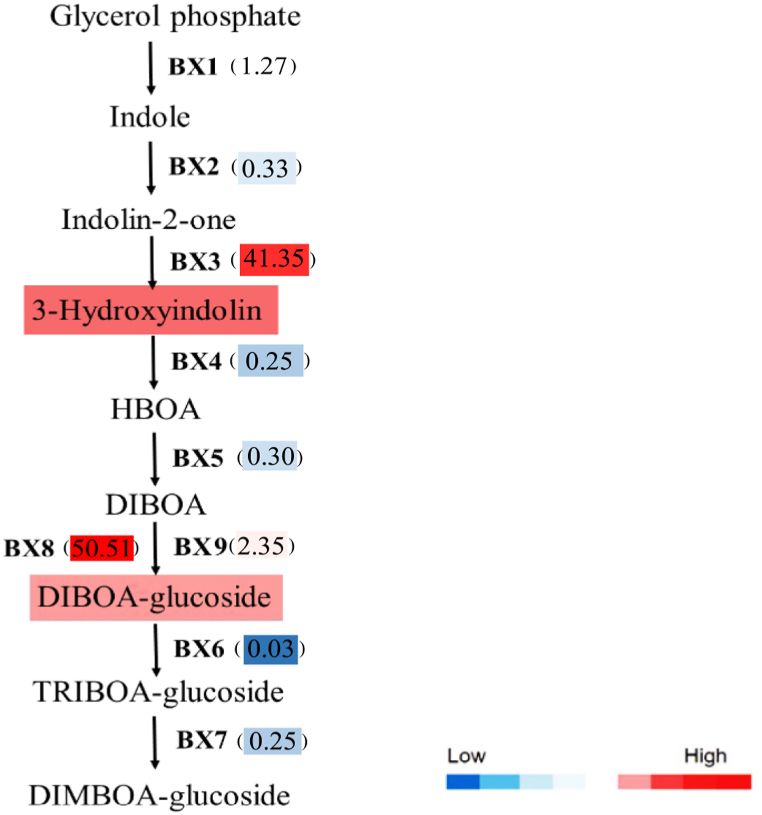
Expression patterns of caterpillar feeding induced genes and metabolites involved in benzoxazinoid biosynthesis. Values in brackets are presented genes expression fold change relative to untreated control. Different colors represent gene expression levels.

To protect themselves from herbivores, plants synthesize various toxic metabolites. Bxs are among the most important plant defense compounds in many plants, including wheat and maize [33]. Specifically, we focused on the transcriptional levels of *Bx* genes which encode the key enzymes for the Bxs biosynthesis. The transcriptional abundance of *Bx1*, which encodes the first enzyme in the Bxs biosynthesis only exhibited a slight increase. Some genes such as *Bx3* and *Bx8/9* were significantly induced by *S. exigua* attack, whose enconding products catalyze biosynthesis of 3-hydroxy-indolin and 2-β-D-glucopyranosyloxy-4-hydroxy-1,4-benzoxazin-3-one (DIBOA-Glc). As expected, the contents of metabolites 3-hydroxy-indolin and DIBOA-Glc were notably enhanced. Others encoding *Bx2*, *Bx4*, *Bx5*, *Bx6* and *Bx7* were dramatically downregulated (Fig. 2). Due to their low abundance, transcripts of *Bx10/11*, *Bx12/14* and *Bx13* could not be detected in the transcriptome data.

### 3.9 Plant hormone-related genes induced by *S. exigua* damage

The hormone-related genes in maize were induced by *S. exigua* damage (Table S7). SA (salicylic acid)-related genes including three transcripts of salicylic acid-binding protein 2 were up regulated by *S. exigua* feeding, while the *PAL* gene involving in SA biosynthesis was down regulated. There were five JA-related genes detected to be differentially expressed, among which, genes encoding jasmonic acid-amido synthetase (JAR1), allene oxide cyclase 3 (AOC3) and lipoxygenase 12 (LOX12) were significantly reduced, the other two genes encoding lipoxygenase 6 (LOX6) and 12-oxophytodienoate reductase 1 (OPR1) were highly induced in insect-resistant maize when subjected to the *S. exigua* attack. Besides, transcriptional abundance of genes associated with other phytohormones exhibited different transcriptional levels, some genes were upregulated and others were downregulated.

### 3.10 Concentrations of JA and SA after *S. exigua* attack

JA and SA play essential parts in plant response against insect herbivory [34,35]. In order to identify whether these two hormones participate plant defense against insect-herbivore damage, the concentrations of the two compounds were determined by HPLC. However, the data indicated that no significant differences in the contents of the two phytohormones were detected before and after *S. exigua* attack.

### 3.11 Transcriptome sequencing data validation using quantitative real-time PCR (qRT-PCR)

The five up-regulated genes, which encoded isovaleryl-CoA dehydrogenase, aldehyde dehydrogenase, amino deoxychorismate synthase, L-aspartate oxidase, and catalase isozyme 2, also exhibited a significant increase in relative expression. In addition, five down-regulated genes encoding 2,3-bisphosphoglycerate-independent phosphoglycerate mutase, threonine dehydratase, 60S ribosomal protein L12, 40S ribosomal protein S27 and triosephosphate isomerase showed similar declining expression trends with a coincidence rate 98.96 % (Table S1), indicating that the transcriptomic result was reliable.

## 4 Discussion

Our present study presented the first effort to combine transcriptomic and metabolic techniques for the comparative analyses of the genes and the metabolites involved in insect-resistant maize response to damage caused by *S. exigua* infestation. Transcriptomic analysis demonstrated that more DEGs were down-regulated than up-regulated in response to *S. exigua* feeding, which was different from previous research focusing on maize under the same treatment (*S. exigua* infestation) [13]. This difference could be explained by the use of differing inbred maize lines in previous research and our study. The former employed inbred line B73, which is highly sensitive to insect infestation, while the insect-resistant inbred line was utilized in the current study. This implied that different response mechanisms might be stimulated between resistant and sensitive inbred lines subjected to caterpillar feeding. In addition, seedling ages, herbivore species, feeding sites, and calculation methods for gene expression might also result in the variability of transcriptomic results [[36], [37], [38]].

Here, our results showed that expression of jasmonic acid-amido synthetase declined after *S. exigua* feeding in resistant CML139 (Table S7). Similarly, JA accumulation was not significantly reduced by *S. exigua* infestation, nor were there remarkable differences between B73 and Ky21 [7], which conflicted with the prior report regarding the increasing accumulation JA in B73 [39]. This disagreement may have resulted from the differing infestation position of B73. Enhanced JA production occurred when the third leaf was fed to *S. exigua*. However, there was no significant accumulation of JA when the second leaf was introduced as the feeding site. Of note, no matter which leaf was infested, no remarkable changes in JA occurred in Ky21 and CML139 [7], both of which are insect-resistant lines, suggesting that maize resistance response for pest defense might not depend on the JA-regulating pathway.

Furthermore, we focused on the transcriptional abundance of the related genes that involve in JA biosynthesis, such as genes encoding allene oxide synthase (AOS), allene oxide cyclase (AOC) and oxophytodienoate reductase (OPR). For instance, *AOS* genes, which encode enzymes catalyzing the second step of the JA pathway, were not detected in CML139 due to their low abundance. In contrast, *AOS* genes in B73 were significantly induced [13]; *AOC3* gene encoding enzyme that catalyzes the third step in JA pathway was decreased dramatically in CML139, which was inconsistent with *AOC* genes in B73 exhibiting a notable enhancement; In addition, the expression levels of the genes encoding OPR which catalyze the fourth step of JA pathway were diverse in different family numbers. *OPR1* was notably enhanced, *OPR2* exhibited a slight increase, whereas *OPR5*, *OPR7* and *OPR11* only decreased a little in CML139 after caterpillar feeding. However, in inbred line B73, *OPR7* was highly induced, *OPR1* and *OPR2* increased slightly, *OPR3* and *OPR6* decreased. The above results indicated that the expression levels of *AOS*, *AOC* and *OPR* genes, which encode key enzymes involving in JA biosynthesis varied between insect-resistant and insect-susceptible inbred lines of maize when subjected to *S. exigua* attack.

Aromatic amino acids in plants serve as precursors for a wide range of secondary metabolites that are important for plant growth and defense [[40], [41], [42]]. An obvious accumulation of amino acids was observed in maize leaves under herbivore attack. Among these amino acids, phenylalanine (Phe), tryptophan (Trp) and tyrosine (Tyr) were all notably induced by *S. exigua* feeding. They function as precursors for defensive metabolites such as benzoxazinoids, phenylpropanoids and terpenoid. Similar results were also reported by previous studies [37,38,43]. This result implied that aromatic amino acids might be important for maize defense against herbivore feeding.

Evidences suggested the role of flavonoids in plant defense against insect pests [44,45]. A similar finding was observed in the present work as five genes encding CYP73A, ARATH Shikimate O-hydroxycinnamoyltransferase (HST), Caffeoyl-CoA 3-O-methyltransferase (ROMT15), CYP98A and CYP75B1 and two metabolites (kaempferol-3-rutinoside and Rutin) involved in flavonoid biosynthesis were significantly induced. However, another study found that decreased gene expression pattern clusters contained genes associated with flavonoid biosynthesis [13]. This contradiction may also be due to the differing use of resistant or susceptible inbred maize lines. Rutin is a common flavonol glycoside present in many plants including buckwheat, passionflower, onion, oranges, apple, lemon, grapes, and tea [46]. Our results identified enhanced rutin accumulation in maize response to *S. exigua* infestation, which was supported by a recent study in which rutin was a promising insecticide candidate for insect pest management [47]. The above result demonstrated that rutin might function as a positive compound in resistant maize response to *S. exigua* feeding.

TFs play an important role in plant response to herbivory [[48], [49], [50]]. In our transcriptomic data, 741 TFs were involved in the *S. exigua* attack response, suggesting that the resistant defense might be a complicated process. The transcriptional level of MYB TF families was the most modulated by *S. exigua* infestation. There have been increasing evidences indicating that MYB TFs could improve plant resistance to insect attack ([51]; Pleet et al., 2010; [52]). It was interesting that four JA-responsive MYB TFs have been characterized to directly repress rutin biosynthesis in *Fagopyrum tataricum* [53]; this is consistent with our results that an enhanced accumulation of rutin occurred while JA and its derivatives exhibited no remarkable inductions at either transcriptional or metabolic level in maize attacked by *S. exigua*. These results implied that there might be an antagonistic interaction between JA and rutin. MYB TFs might play an essential role in this interaction at the transcriptional level.

In plants, phenylpropanoids have been identified to be induced by insect feeding functioning as direct resistance to herbivory [50,54,55]. It is well known that the shikimate pathway is an important pathway in plants and is responsible for the biosynthesis of the aromatic amino acids, auxin, SA, lignin, and phenylpropanoids [41]. In the current study, we found that genes involved in the shikimate pathway such as SK and CM were significantly induced, accompanied with elevated contents of phenylalanine and phenylethylamine, suggesting that the shikimate-mediated secondary metabolism was extremely important during maize resisting against *S. exigua* attack.

When subjected to herbivore feeding, plants produce several aromatic metabolites including phenylethanol, phenylethyl-b-D-glucopyranoside and phenylethylamine that function in direct and indirect plant defense and defense signaling. Our data showed that phenylethylamine was significantly increased upon herbivory, which was consistent with the prominent accumulation of phenylethylamine in poplar upon herbivore attack [55].

Among enzymes involving in phenylethylamine metabolism, PAL is a key enzyme for plant defense against pathogens, but the role of PAL in plant resistance to insect is still poorly understood [56]. Here, we demonstrated that expression level of the *PAL1* gene was dramatically decreased in maize leaves infested by *S. exigua*. However, this result was in inconsistent with the previous studies [38,56,57], in which *PAL* gene expression was induced in both susceptible and resistant rice plants infested by herbivory insects. The contradiction could appear reasonable that *PAL* might function distinctly in different species; another reason might be the remarkably accumulations of both phenylalanine and phenylethylamine in our result. In detail, there are two metabolic branches beginning from phenylalanine. One pathway is that PAL catalyzes phenylalanine to synthesis cinnamic acid; the other is that phenylethylamine is produced by phenylalanine. The decreased transcriptional abundance of the *PAL1* gene leaded to the accumulation of phenylalanine, serving as the substrate of phenylethylamine. The similar result was also achieved in the previous report, in which, phenylalanine hyperaccumulation could be achieved by mutations in *PAL* [58].

To date, researches have demonstrated that Bxs possess a wide range of beneficial properties including insecticidal, antifungal and anti-microb [33]. Therefore, we investigated expressional profiles of *Bx* genes in response to *S. exigua* feeding. Among them, *Bx3* and *Bx8/9* were highly induced, whereas there was a mild increase in the transcript of *Bx1*. In contrast, *Bx2*, *Bx4*, *Bx5*, *Bx6* and *Bx7* expressions were sharply decreased after 24 h of caterpillar infestation. Additionally, transcriptional values of the rest genes involved in Bxs biosynthesis were not obtained from RNA-seq data because of their low abundances. These results were partly distinguished from the recent report [13], in which transcriptional levels of all *Bx* genes were notably enhanced after 24 h of *S. exigua* feeding. The inconsistence could be explained that different inbred lines might trigger different *Bx* genes when subjected to *S. exigua* attack.

Previous researches demonstrated that the contents of Bxs were strongly increased in maize by herbivore attack.In B73, which is susceptive to insect feeding, both DIMBOA-Glc and HDMBOA-Glc abundances gradually increase from 4 to 24 h of caterpillar infestation [13]; Whereas only HDMBOA-Glc content was remarkably induced in another insect-resistant line Ky21 by *S. exigua* feeding [7]. Intriguingly, our result showed there was only DIBOA-Glc concentration significantly increased in the insect-resistant line CML139. The inconformity might sound reasonable likely because there is a great variation in Bxs abundance among different maize inbred lines [59]. These results confirmed Bxs biosynthesis is not the only defense mechanism induced by caterpillar feeding on maize plants.

Although DIBOA-Glc has not been reported as defense-related Bxs of maize in the previous researches, it was identified as an important secondary metabolite for defense in plants. For instance, DIBOA-Glc was identified as the atrazine-degrading compound present in the roots of Eastern gamagrass {*Tripsacum dactyloides* (L.)} [60]. Furthermore, the concentration of DIBOA-Glc was significantly elevated in the maize leaves treated by *Rhizoctonia solani* [61]. Therefore, DIBOA-Glc might be another active Bxs compound response to herbivore infestation in the insect–resistant inbred line, which need require further investigations.

## 5 Conclusions

Overall, the present study examined insect-resistant inbred line of maize seedlings in response to damage caused by *S. exigua* feeding through integrated transcriptomic and metabolomic analyses. The integrative analysis demonstrated that the defense response was mainly associated with amino acids, phenylpropanoids and benzoxazinoids, with exception of JA, SA and their derivatives. Further exploration of the metabolites induced by *S. exigua* infestation should be carried out in order to improve maize cultivar breeding with enhanced resistance to *S. exigua*. Taken together, our results provided new insights into the molecular mechanisms underlying maize resistant defense upon insect herbivory.

## CRediT authorship contribution statement

**Qiulan Luo:** Writing – review & editing, Writing – original draft, Investigation, Funding acquisition, Data curation, Conceptualization. **Fangmeng Duan:** Visualization, Methodology, Investigation, Data curation, Conceptualization. **Wenwen Song:** Writing – original draft, Formal analysis, Data curation, Conceptualization.

## Data availability statement

The data presented in this work are available in the article and Supplementary Materials.

## Funding

This research was funded by 10.13039/501100003453Natural Science Foundation of Guangdong Province (2024A1515010278), China; Guangdong Provincial Key Laboratory of Functional Substances in Medicinal Edible Resources and Healthcare Products (2021B1212040015),China; Guangdong Provincial Department of Education's Characteristic Innovation Project (2023KTSCX084), China; doctoral scientific research foundation of 10.13039/501100010844Hanshan Normal University (QD202120, E22049), China.

## Declaration of competing interest

We declare that we have no financial and personal relationships with other people or organizations that can inappropriately influence our work, there is no professional or other personal interest of any nature or kind in any product, service and/or company that could be construed as influencing the position presented in, or the review of, the manuscript entitled.
