# Supplementary material for: Transcriptomics integrated with metabolomics reveals the defense response of insect-resistant Zea mays infested with Spodoptera exigua
Source: Heliyon. 2025 Feb 8;11(4):e42565. doi: 10.1016/j.heliyon.2025.e42565 (PMC11872508; doi:10.1016/j.heliyon.2025.e42565)
Supplement: Multimedia component 1 [file mmc1.docx]

Table S1. The validation of transcriptome sequencing data using qRT-PCR

| Gene id | Annotation information | Primer sequence (5'-3') | Product size/bp | Annealing  temperature/°C | Fold change (log 2) | |
| --- | --- | --- | --- | --- | --- | --- |
|  |  |  |  |  | RNA-seq | qRT-PCR |
| 100217040 | Isovaleryl-CoA dehydrogenase | F: AGGAGGTGAGGGCGATTTA  R: ATTTGATGTATGTCTGCGGTTAT | 81 | 57 | 1.62 | 2.47 |
| 103641646 | Aldehyde dehydrogenase | F: ACCCAGACTTCAGACTCCG  R: CTTCTCGTCCACCACCAGC | 253 | 59 | 0.97 | 1.26 |
| 103629593 | Aminodeoxychorismate synthase | F: CACCTTCCTTCCGCCTACGC  R: TGACGACCGACAGCTCCTGG | 178 | 64 | 0.92 | 1.48 |
| 103644300 | L-aspartate oxidase | F: CGCACGACACCGACTAAG  R: GACCAACCACGCCCTACT | 164 | 55 | 2.19 | 3.48 |
| 542230 | Catalase isozyme 2 | F: GGACCCAGCAAGGCAAGA  R: GGAAACCCAGCCAAACAT | 467 | 58 | 0.87 | 1.12 |
| 103651775 | 2,3-bisphosphoglycerate-independent phosphoglycerate mutase | F: GGTGGTGGAAGGATGTATG  R: GGCTCGGAAGTTGATAGTG | 255 | 58 | -1.04 | -1.31 |
| 103625947 | Threonine dehydratase | F: CGCACATTCATACCTCCCT  R: AGCGTCTCGACTGACCATAA | 360 | 56 | -0.67 | -1.52 |
| 100281960 | 60S ribosomal protein L12 | F: ACCGCAAGAAGGTCAAGAACA  R: GGCTTCAGCAGCAAAGATAACAC | 271 | 62 | -1.05 | -2.07 |
| 103627157 | 40S ribosomal protein S27 | F: AATGATGAGCCAGCAGGTTG  R: GAGTCTGAGCAGGGAGGGTT | 89 | 58 | -1.78 | -2.19 |
| 100382908 | Triosephosphate isomerase | F: CAATGGGTTATTCTTGGACACTCTG  R: TTGATGACCTTGACTGGCTTC | 329 | 62 | -1.79 | -2.76 |
