# Supplementary material for: Transcriptomics integrated with metabolomics reveals the defense response of insect-resistant Zea mays infested with Spodoptera exigua
Source: Heliyon. 2025 Feb 8;11(4):e42565. doi: 10.1016/j.heliyon.2025.e42565 (PMC11872508; doi:10.1016/j.heliyon.2025.e42565)
Supplement: Multimedia component 2 [file mmc2.docx]

Table S2. Summary of sequencing data quality

| Sample | Raw reads | Clean reads | Clean bases | Error rate (%) | Q20 (%) | Q30 (%) | GC content (%) |
| --- | --- | --- | --- | --- | --- | --- | --- |
| Treat1 | 50,080,070 | 48,164,138 | 7.22G | 0.02 | 96.38 | 90.94 | 57.97 |
| Treat2 | 56,904,748 | 55,368,042 | 8.31G | 0.02 | 96.40 | 90.84 | 57.27 |
| Treat3 | 56,694,536 | 54,628,752 | 8.19G | 0.02 | 96.70 | 91.59 | 57.50 |
| CK1 | 41,793,746 | 40,349,918 | 6.05G | 0.02 | 96.72 | 91.63 | 57.52 |
| CK2 | 45,927,578 | 44,158,402 | 6.62G | 0.02 | 96.68 | 91.55 | 57.47 |
| CK3 | 54,226,448 | 52,277,572 | 7.84G | 0.02 | 96.66 | 91.50 | 57.91 |

Note: The treatment was 24 hours after caterpillars introduced to the plants. CK means un-infested leaves of healthy seedlings; Treat means caterpillar-treated plants.
