# Supplementary material for: Transcriptomics integrated with metabolomics reveals the defense response of insect-resistant Zea mays infested with Spodoptera exigua
Source: Heliyon. 2025 Feb 8;11(4):e42565. doi: 10.1016/j.heliyon.2025.e42565 (PMC11872508; doi:10.1016/j.heliyon.2025.e42565)
Supplement: Multimedia component 5 [file mmc5.docx]

Table S5. The list of differentially abundant metabolites

| Metabolites | log2 FC | *P* value | Metabolite | log2 FC | *P* value |
| --- | --- | --- | --- | --- | --- |
| salicylate | 2.63 | 4.21E-03 | Hydroxycitric acid | 0.93 | 6.75E-05 |
| L-threonine | 2.52 | 1.16E-03 | deethylatrazine | 0.93 | 1.60E-04 |
| D-aspartic acid | 2.50 | 2.54E-07 | L-asparagine | 0.71 | 2.47E-02 |
| L-proline | 2.36 | 3.94E-03 | L-tyrosine | 0.69 | 1.42E-02 |
| choline | 2.30 | 1.05E-02 | beta-alanine | 0.66 | 2.64E-02 |
| L-Norvaline | 2.25 | 7.51E-05 | kaempferol-3-rutinoside | 0.65 | 1.91E-02 |
| L-serine | 2.14 | 7.43E-04 | 2-phenylethylamine | 0.53 | 8.12E-05 |
| piperidine | 2.13 | 4.42E-03 | L-tryptophan | 0.51 | 8.16E-04 |
| L-methionine | 2.12 | 1.83E-05 | tyramine | 0.49 | 3.09E-03 |
| 3-Hydroxyindolin | 2.11 | 1.81E-03 | N-methylnicotinate | 0.22 | 2.05E-02 |
| DIBOA-glucoside | 1.85 | 1.92E-02 | taurine | -0.23 | 2.74E-03 |
| L-leucine | 1.64 | 3.28E-04 | 2-methylpropanal oxime | -0.40 | 4.14E-03 |
| Rutin hydrate | 1.55 | 2.28E-03 | citric acid | -1.09 | 3.09E-02 |
| L-isoleucine | 1.50 | 4.13E-06 | guanosine | -1.32 | 2.01E-02 |
| L-histidine | 1.44 | 1.83E-02 | mesaconate(2-) | -1.36 | 6.23E-04 |
| D-phenylalanine | 1.28 | 5.65E-05 | cis-aconitate(3-) | -1.39 | 4.58E-04 |
| shikimate | 1.21 | 3.12E-03 | 2-oxoglutaric acid | -2.85 | 2.84E-09 |
