# Supplementary material for: Transcriptomics integrated with metabolomics reveals the defense response of insect-resistant Zea mays infested with Spodoptera exigua
Source: Heliyon. 2025 Feb 8;11(4):e42565. doi: 10.1016/j.heliyon.2025.e42565 (PMC11872508; doi:10.1016/j.heliyon.2025.e42565)
Supplement: Multimedia component 7 [file mmc7.docx]

Table S7. Hormone-related genes induced by *S. exigua* damage

| Gene ID | Gene annotation details | log2 fold change |
| --- | --- | --- |
| Salicylic acid related genes | | |
| 103636584 | Salicylic acid-binding protein 2 | 2.4061 |
| 103641536 | Salicylic acid-binding protein 2 | 1.5245 |
| 103651128 | Salicylic acid-binding protein 2 | 4.3249 |
| 100384215 | Phenylalanine ammonia-lyase PAL | -2.7509 |
| Jasmonic acid related genes | | |
| 100381950 | Jasmonic acid-amido synthetase JAR1 | -0.8179 |
| 100273356 | Allene oxide cyclase 3 AOC3 | -2.4083 |
| 100037806 | lipoxygenase 6 LOX6 | 1.2544 |
| 100037829 | lipoxygenase 12 LOX12 | -1.2676 |
| 732815 | 12-oxophytodienoate reductase 1 OPR1 | 3.1008 |
| Abscisic acid related genes | | |
| 100191697 | Abscisic acid hydroxylase 3 | -1.9152 |
| 103647484 | Abscisic acid hydroxylase 3 | 4.2211 |
| 100274679 | Abscisic acid receptor PYL10 | 2.1325 |
| 103646020 | Abscisic acid receptor PYL10 | -1.2748 |
| 100383950 | Abscisic acid receptor PYL2 | 1.1204 |
| 100281868 | Abscisic acid receptor PYL4 | 2.8531 |
| 103634514 | Abscisic acid receptor PYL4 | 1.6798 |
| 100273694 | Abscisic acid receptor PYL8 | -1.1008 |
| 100382238 | Abscisic acid receptor PYR1 | 1.8977 |
| 100194217 | abscisic acid-inducible protein | -2.5928 |
| 542562 | abscisic acid-inducible protein | -3.1179 |
| 542725 | abscisic acid-inducible protein | -0.6082 |
| 100501609 | Abscisic acid-insensitive 5-like protein 2 | 1.0584 |
| 100191211 | Abscisic acid-insensitive 5-like protein 3 | -4.0483 |
| 100502540 | Abscisic acid-insensitive 5-like protein 4 | 1.8939 |
| 100194287 | Abscisic acid-insensitive 5-like protein 7 | 1.8601 |
| Auxin related genes | | |
| 100857063 | Auxin response factor 1 | -1.6469 |
| 103637942 | Auxin response factor 16 | 2.2766 |
| 100502480 | Auxin response factor 17 | -2.0873 |
| 100384045 | Auxin response factor 2 | -1.1505 |
| 103642400 | Auxin response factor 21 | 1.1528 |
| 100273544 | Auxin response factor 24 | -0.8878 |
| 100383196 | Auxin response factor 24 | -1.3113 |
| 100279854 | Auxin response factor 25 | -1.0616 |
| 100383168 | Auxin response factor 3 | -1.2183 |
| 100274571 | Auxin response factor 4 | 0.8420 |
| 103626605 | Auxin response factor 5 | -1.2231 |
| 103653810 | Auxin response factor 8 | 1.4966 |
| 103630153 | Auxin response factor 9 | 1.6673 |
| 103646418 | Auxin response factor 9 | 1.4878 |
| 103643338 | Auxin-induced in root cultures protein 12 | -1.6689 |
| 103653191 | Auxin-induced in root cultures protein 12 | 3.8239 |
| 100272328 | Auxin-induced protein 5NG4 | -1.0517 |
| 100193444 | Auxin-responsive protein IAA1 | 2.1939 |
| 100194253 | Auxin-responsive protein IAA1 | 2.9382 |
| 100216870 | Auxin-responsive protein IAA12 | -4.9827 |
| 100274141 | Auxin-responsive protein IAA12 | -3.8178 |
| 100284262 | Auxin-responsive protein IAA13 | -1.8861 |
| 100274569 | Auxin-responsive protein IAA17 | -2.5068 |
| 100272504 | Auxin-responsive protein IAA18 | -1.6733 |
| 100274580 | Auxin-responsive protein IAA2 | 3.7753 |
| 100272577 | Auxin-responsive protein IAA23 | 1.1669 |
| 100384587 | Auxin-responsive protein IAA31 | -2.7902 |
| 103634643 | Auxin-responsive protein IAA4 | -1.1411 |
| 103650054 | Auxin-responsive protein IAA4 | 1.4487 |
| 100284457 | Auxin-responsive protein IAA5 | -0.8954 |
| 100283620 | Auxin-responsive protein IAA6 | 2.2349 |
| 100281448 | Auxin-responsive protein IAA9 | 1.7942 |
| 107522023 | Auxin-responsive protein IAA9 | 3.9415 |
| 100279159 | Auxin-responsive protein SAUR32 | -1.2077 |
| 100281944 | Auxin-responsive protein SAUR36 | -2.5733 |
| 100283813 | Auxin-responsive protein SAUR36 | -4.0822 |
| 103627479 | Auxin-responsive protein SAUR36 | 2.1056 |
| 103646147 | Auxin-responsive protein SAUR36 | 2.8149 |
| 103643781 | Auxin-responsive protein SAUR71 | -2.8367 |
| 100272759 | Probable auxin efflux carrier component 3a | -2.5783 |
| 109941620 | Probable auxin efflux carrier component 3a | -2.0912 |
| 100281763 | Probable auxin efflux carrier component 6 | -0.9514 |
| Cytokinin related genes | | |
| 542507 | Cytokinin dehydrogenase 4 | 2.9427 |
| 100272805 | Cytokinin dehydrogenase 5 | 3.5067 |
| 100272861 | Probable cytokinin riboside 5, monophosphate phosphoribohydrolase LOGL9 | -0.8873 |
| 100282510 | Probable cytokinin riboside 5, monophosphate phosphoribohydrolase LOGL10 | 1.2811 |
| 100282620 | Probable cytokinin riboside 5, monophosphate phosphoribohydrolase LOGL4 | -2.4608 |
| 100285847 | Probable cytokinin riboside 5, monophosphate phosphoribohydrolase LOGL7 | 1.2107 |
| 100383171 | Probable cytokinin riboside 5, monophosphate phosphoribohydrolase LOGL10 | -3.6172 |
| Ethylene related genes | | |
| 100192647 | 24-methylenesterol C-methyltransferase 2 | -2.4458 |
| 100282753 | 24-methylenesterol C-methyltransferase 2 | -1.5996 |
| 100384337 | AP2-like ethylene-responsive transcription factor At1g16060 | 2.9874 |
| 103627030 | AP2-like ethylene-responsive transcription factor SNZ | -1.1672 |
| 100273392 | Carboxymethylenebutenolidase homolog | -3.6904 |
| 100279789 | Ethylene insensitive 3-like 1 protein | 0.5977 |
| 100383809 | Ethylene receptor | -1.1408 |
| 103627330 | Ethylene-responsive 3-like 5 protein | 1.6119 |
| 100284048 | Ethylene-responsive transcription ERF010 | 0.8005 |
| 100279803 | Ethylene-responsive transcription factor 1 | 0.8647 |
| 100273194 | Ethylene-responsive transcription factor 12 | 2.7602 |
| 100280582 | Ethylene-responsive transcription factor 4 | 2.5789 |
| 100283393 | Ethylene-responsive transcription factor 4 | 1.0862 |
| 100384218 | Ethylene-responsive transcription factor 4 | 0.8137 |
| 100284527 | Ethylene-responsive transcription factor 8 | 1.6038 |
| 103642291 | Ethylene-responsive transcription factor ABR1 | 3.2193 |
| 100286032 | Ethylene-responsive transcription factor CRF4 | -1.6294 |
| 103654172 | Ethylene-responsive transcription factor ERF003 | -3.3477 |
| 100280932 | Ethylene-responsive transcription factor ERF008 | 2.1822 |
| 103629531 | Ethylene-responsive transcription factor ERF008 | 1.5769 |
| 103638017 | Ethylene-responsive transcription factor ERF008 | 0.97198 |
| 100281327 | Ethylene-responsive transcription factor ERF021 | -0.9081 |
| 103638592 | Ethylene-responsive transcription factor ERF043 | -4.2996 |
| 109939235 | Ethylene-responsive transcription factor ERF053 | 2.2151 |
| 100272581 | Ethylene-responsive transcription factor ERF060 | -1.4923 |
| 103637851 | Ethylene-responsive transcription factor ERF060 | -1.3774 |
| 542569 | Ethylene-responsive transcription factor ERF060 | -2.808 |
| 100192457 | Ethylene-responsive transcription factor ERF073 | 1.6752 |
| 100275504 | Ethylene-responsive transcription factor ERF110 | 1.5973 |
| 100280929 | Ethylene-responsive transcription factor ERF110 | 2.4633 |
| 100275603 | Ethylene-responsive transcription factor ERF113 | 1.3358 |
| 103646542 | Ethylene-responsive transcription factor ERF113 | 2.1570 |
| 100383396 | Ethylene-responsive transcription factor RAP2-11 | -2.9533 |
| 100280553 | Ethylene-responsive transcription factor RAP2-4 | -0.5570 |
| 103645181 | Ethylene-responsive transcription factor RAP2-6 | 2.9774 |
| 103650960 | Ethylene-responsive transcription factor RAP2-6 | 1.7250 |
| 100285135 | Ethylene-responsive transcription factor-like protein At4g13040 | 1.1048 |
| 541794 | Methylenetetrahydrofolate reductase 1 | -1.1497 |
| Indole-3-acetic acid related genes | | |
| 103642161 | Indole-3-acetic acid-induced protein ARG7 | 3.2425 |
| 103633549 | Probable indole-3-acetic acid-amido synthetase GH3.11 | -2.6108 |
| 100193303 | Probable indole-3-acetic acid-amido synthetase GH3.5 | 0.8325 |
| 100280367 | Probable indole-3-acetic acid-amido synthetase GH3.5 | -0.8481 |
| 100381512 | Probable indole-3-acetic acid-amido synthetase GH3.5 | 1.6938 |
| 100280445 | Probable indole-3-acetic acid-amido synthetase GH3.8 | 4.2063 |
