# Supplementary material for: Transcriptomics integrated with metabolomics reveals the defense response of insect-resistant Zea mays infested with Spodoptera exigua
Source: Heliyon. 2025 Feb 8;11(4):e42565. doi: 10.1016/j.heliyon.2025.e42565 (PMC11872508; doi:10.1016/j.heliyon.2025.e42565)
Supplement: Multimedia component 8 [file mmc8.pdf]

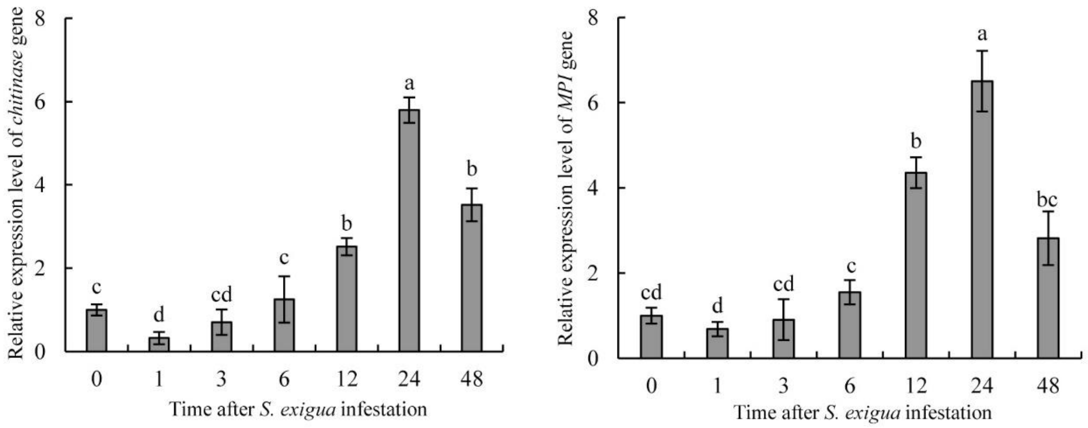

Figure S1. The time expression pattern of chitinase and *MPI* in maize leaves after *S. exigua* feeding. Error bars represent standard deviations (SD) (n=3 biological replicates). Different lowercase letters indicate significant differences at $P < 0.05$.
