# Supplementary material for: Transcriptomics integrated with metabolomics reveals the defense response of insect-resistant Zea mays infested with Spodoptera exigua
Source: Heliyon. 2025 Feb 8;11(4):e42565. doi: 10.1016/j.heliyon.2025.e42565 (PMC11872508; doi:10.1016/j.heliyon.2025.e42565)
Supplement: Multimedia component 10 [file mmc10.pdf]

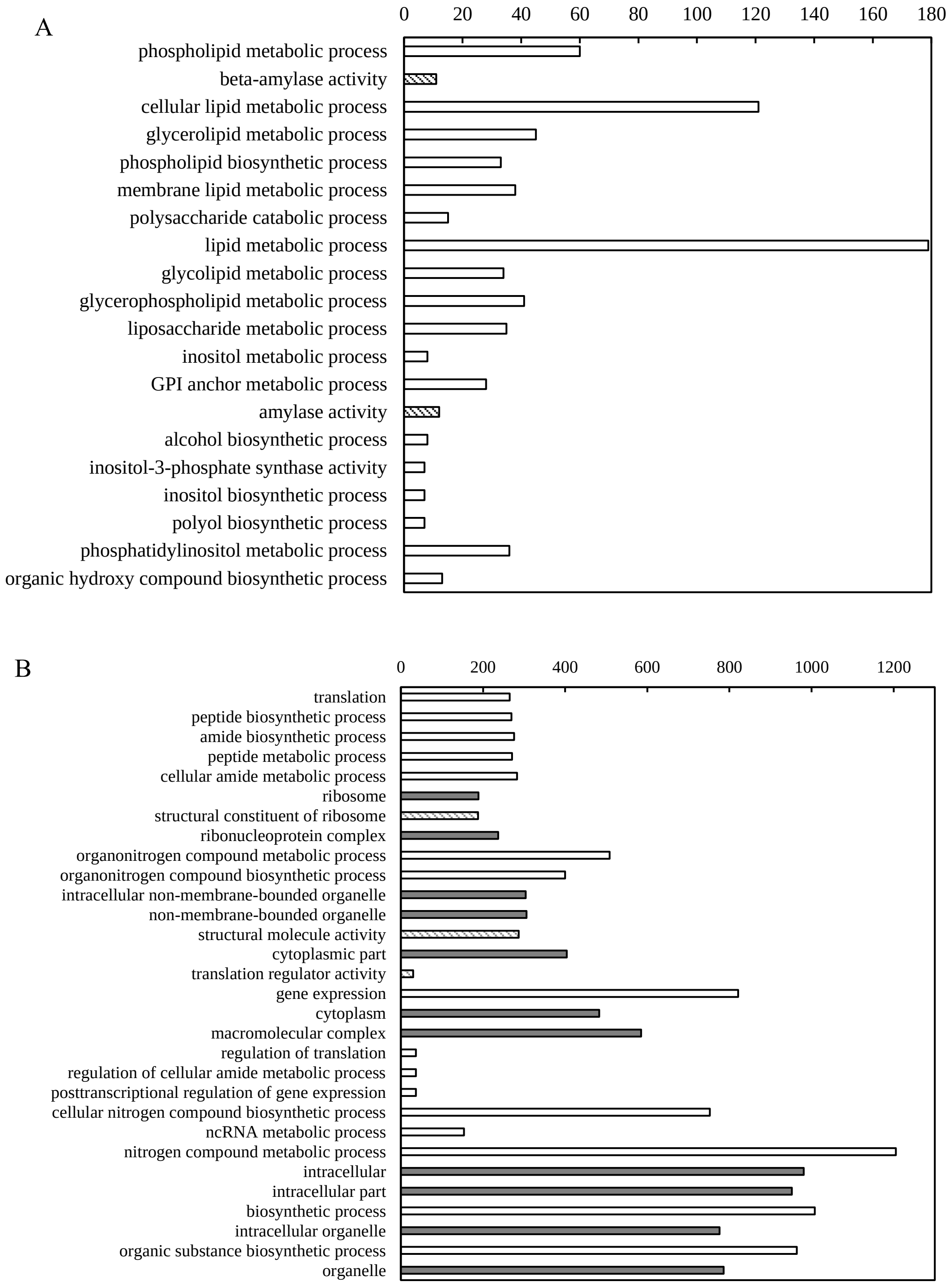

Figure S3. GO analysis of DEGs. (A) The GO analysis of upregulated DEGs. (B) The GO analysis of downregulated DEGs. DEGs in three main GO categories: biological process, molecular function, and cellular component were presented as light, diagonal and grey bars, respectively.
