# Supplementary material for: Transcriptomics integrated with metabolomics reveals the defense response of insect-resistant Zea mays infested with Spodoptera exigua
Source: Heliyon. 2025 Feb 8;11(4):e42565. doi: 10.1016/j.heliyon.2025.e42565 (PMC11872508; doi:10.1016/j.heliyon.2025.e42565)
Supplement: Multimedia component 11 [file mmc11.pdf]

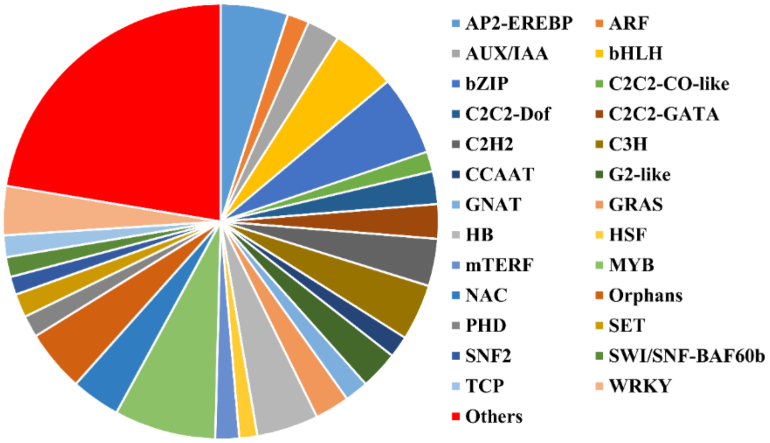

Figure S4. Transcription factor (TF) families in DEGs. The names of TF families were listed on the right. 'Others' contains the number less 10 of TF families.
